# Template‐Free Electrochemical Formation of Silicon Nanotubes from Silica

**DOI:** 10.1002/advs.202001492

**Published:** 2020-07-10

**Authors:** Wei Weng, Jiarong Yang, Jing Zhou, Dong Gu, Wei Xiao

**Affiliations:** ^1^ College of Chemistry and Molecular Sciences Hubei Key Laboratory of Electrochemical Power Sources Wuhan University Wuhan 430072 P. R. China; ^2^ School of Resource and Environmental Sciences Hubei International Scientific and Technological Cooperation Base of Sustainable Resource and Energy Wuhan University Wuhan 430072 P. R. China; ^3^ The Institute of Advanced Studies Wuhan University Wuhan 430072 P. R. China

**Keywords:** electrochemistry, molten salt, silica, silicon nanotubes, silver chloride

## Abstract

Silicon, with its elaborate microstructure, plays important roles in energy materials. In operando engineering of microstructure during extraction is an ideal protocol to develop advanced Si‐based materials. A template‐free electrochemical preparation of silicon nanotubes (Si‐NT) is herein achieved by co‐electrolysis of SiO_2_ and AgCl in molten NaCl–CaCl_2_ at 850 °C. The in situ electrodeposited Ag facilitates the generation of a liquid Ag–Si intermediate, triggering a liquid–solid mechanism to direct the growth of Si‐NT. An automatic separation of Ag from Si then occurs in the following cooling process, resulting in Ag deposits on the Ni current collector and recycling of Ag. Such a facile and smart preparation of Si‐NT from affordable silica guarantees an enhanced current efficiency of 74%, a decreased energy consumption of 12.1 kW h kg_Si_^−1^, and enhanced lithium‐storage capability of the electrolytic Si‐NT. An in situ coating of Ag over the Si‐NT can also be fulfilled by simply introducing soluble AgCl in the melts. The present study provides a template‐free preparation and an in situ surface modification of Si‐NT.

## Graphical abstract

A template‐free preparation and in situ surface modification of silicon nanotubes is achieved by electrochemical reduction of SiO_2_ and AgCl in NaCl–CaCl_2_ molten salt. The elaborately designed process ensures enhanced production yield for massive Si extraction and enhanced lithium‐storage capability of the electrolytic Si.

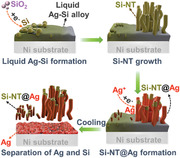

With the rapid development of electric vehicles and large‐scale electrical energy storage systems, Li‐ion batteries (LIBs) with high energy and appealing cycling stability are in urgent need.^[^ ^1^, ^2^, ^3^ ^]^ Silicon (Si) is regarded as one of the most promising candidates as anode materials in LIBs because of high specific capacity and low working potential.^[^ ^4^, ^5^, ^6^ ^]^ The theoretical specific capacity of Si anode is high up to 4200 mAh g^−1^, ten times higher than the state‐of‐the‐art graphite anode.^[^ ^7^, ^8^ ^]^ The low working potential of Si anode guarantees high voltage and energy density when equipped in full cells.^[^ ^8^ ^]^ However, the large volume variation (>300%) during lithiation/delithiation process and low intrinsic conductivity of Si anode cause a rapid capacity fading and poor high‐rate performance,^[^ ^7^, ^8^, ^9^ ^]^ significantly retarding the large‐scale deployment of Si anode.

Silicon nanotubes (Si‐NT) represent a promising structure to mitigate the aforenamed challenges.^[^ ^3^, ^4^, ^10^ ^]^ The free empty space inside the tube can accommodate more volume changes, provide extra electrolyte‐accessible inner surfaces, and guarantee shortened diffusion length for lithium ions.^[^ ^2^, ^4^, ^5^ ^]^ In addition, the 1D character of Si‐NT can facilitate axial charge transfer and shorten the radial Li‐ion diffusion distance.^[^ ^11^ ^]^ Coating a high‐conductivity surface layer can further improve the performance of Si‐NT as anode materials because such a surface layer can not only maintain the structure integrity of Si nanostructures by resisting the volume variation during the lithiation/delithiation process, but also increase the conductivity of Si.^[^ ^12^, ^13^, ^14^, ^15^, ^16^ ^]^

Currently, Si‐NT are mainly prepared by chemical vapor deposition (CVD) or template‐assisted methods.^[^ ^12^, ^17^, ^18^, ^19^, ^20^, ^21^, ^22^, ^23^, ^24^, ^25^, ^26^, ^27^, ^28^ ^]^ However, the usage of toxic SiH_4_/SiCl_4_ as raw materials and expensive gold as catalysts as well as the low production yield largely restrict the large‐scale deployment of the CVD methods.^[^ ^19^, ^20^, ^21^, ^23^ ^]^ The template‐assisted strategy requires tedious procedures including the construction of sacrificial templates, coating of Si‐containing precursors, extraction of Si, and removal of inner template.^[^ ^12^, ^22^, ^23^, ^24^, ^25^, ^26^, ^27^, ^28^ ^]^ The preparation procedures become more complicated when an extra surface layer is coated via post‐processing strategies to improve the performance of Si‐NT.^[^ ^11^, ^29^, ^30^ ^]^ Exploring practical methods for large‐scale and low‐cost preparation of Si‐NT from affordable silicon sources as well as in situ coating strategy is an appealing but tough task.

Molten salt electrolysis has been proven to be an effective way for large‐scale conversion of affordable silica (SiO_2_) to solid Si nanostructures at temperatures lower than 900 °C.^[^ ^31^, ^32^, ^33^, ^34^, ^35^, ^36^, ^37^, ^38^, ^39^, ^40^, ^41^ ^]^ However, the solid‐solid conversion from SiO_2_ to Si makes the nucleation‐growth process of Si hardly tunable, therefore construction of hollow Si nanostructure by molten salt electrolysis of silica is an insurmountable challenge.^[^ ^31^, ^32^, ^33^, ^34^, ^35^, ^36^, ^37^, ^38^, ^39^, ^40^, ^41^ ^]^ As for coating a conductive buffer layer on the surface of Si nanostructures, carbon is commonly used as the candidate.^[^ ^12^, ^13^, ^14^, ^15^, ^16^ ^]^ However, the unpleasant SiC is readily generated during molten salt electrolysis, therefore making the in situ coating strategy far from expectations.^[^ ^11^ ^]^

Herein, template‐free electrochemical formation of Si‐NT from SiO_2_ and in situ coating of an Ag buffer layer over the Si‐NT are realized via the co‐electrolysis of SiO_2_ and AgCl in molten NaCl–CaCl_2_ at 850 °C. As illustrated in **Figure** **1**, Ag is first electrodeposited on the Ni current collector (step 1), followed by the subsequent reduction of SiO_2_ to Si for formation of liquid Ag–Si alloy (step 2). The continuous reduction of SiO_2_ to Si and dissolution of Si into the liquid Ag–Si results in the oversaturation of Si, contributing to the precipitation of Si‐NT via the classical liquid‐solid mechanism (step 3).^[^ ^20^, ^21^ ^]^ The oversaturated Si prefers to precipitate along the high‐energy edges of the liquid Ag–Si cluster, facilitating the formation of Si‐NT.^[^ ^20^, ^21^ ^]^ Specially, Ag is preferentially adhered to the Ni substrate due to a strong interaction between Ag and Ni, resulting in a self‐separation of Ag from Si during the cooling process of liquid Ag–Si alloy. This automatic separation contributes to recovery of Ag and generation of Si‐NT powder (step 4). By dissolving excessive AgCl into the molten salts, Ag can be in situ coated on the surfaces of Si‐NT, providing an in situ surface modification of Si‐NT. The present protocol shows a high current efficiency (74%) and low energy consumption (12.1 kWh kg_Si_^−1^, lower than that of commercial metallurgical grade silicon), promising practical applications on massive production of advanced Si applicable to LIBs.

**Figure 1:**
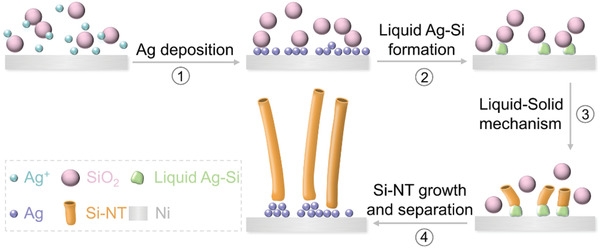
Illustration on the formation mechanism of Si‐NT.

Experimental details are provided in the Supporting Information. The control experiments are performed by using different solid cathodes and a graphite anode in molten NaCl–CaCl_2_ (equal in molar) at 850 °C. Solid SiO_2_, SiO_2_–Ag mixture, and SiO_2_–AgCl mixture are used as the solid cathodes, which are all sandwiched by Ni or Mo meshes. The Si/Ag molar ratios in the latter two cases are controlled to be 10. **Figure** **2**a compares the cyclic voltammetry (CV) behaviors of AgCl (blue curve), SiO_2_ (cyan curve) and SiO_2_–AgCl (red curve) powders in the melt by using a home‐made Ag/AgCl as the reference electrode. The electrochemical reduction of AgCl (blue curve) and SiO_2_ (cyan curve) peak at 0 V (versus Ag/AgCl) and −0.4 V (versus Ag/AgCl) respectively, as evidenced by the comparison between the CV curves and the background (grey curve). The results show the preferential reduction of AgCl than that of SiO_2_.^[^ ^42^, ^43^ ^]^ For the CV curve of SiO_2_–AgCl mixture (red curve), the reduction peak at −0.4 V (versus Ag/AgCl) is distinctly broadened. The enlarged reduction peak is attributed to the overlapping between formation of Si and Ag–Si, which is further manifested by the newly formed oxidation peak at 0.2 V (versus Ag/AgCl) due to the oxidation of Ag–Si.^[^ ^43^ ^]^

**Figure 2:**
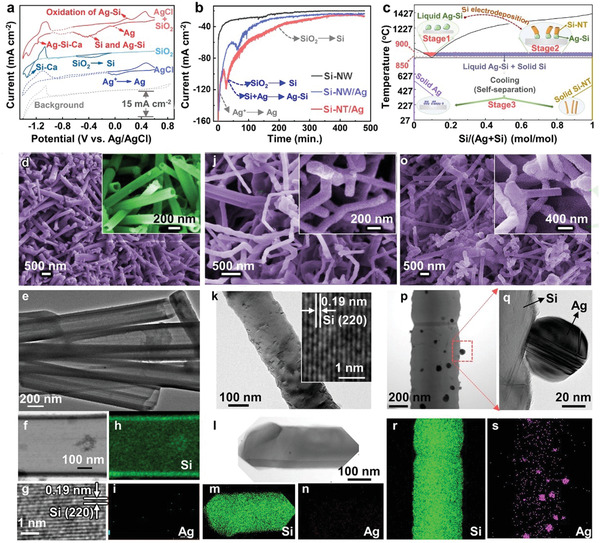
a) CV curves of Mo (background), AgCl‐loaded Mo, SiO_2_‐loaded Mo, and AgCl–SiO_2_‐loaded Mo electrodes recorded at a scan rate of 5 mV s^−1^ in molten NaCl–CaCl_2_ at 850 °C; b) current–time curves for the constant‐voltage electrolysis of SiO_2_ (black curve), SiO_2_–Ag mixture (blue curve) and SiO_2_–AgCl mixture (red curve); and c) Ag–Si phase diagram. d–s) Characterizations of Si‐NT/Ag obtained from the electrolysis of solid SiO_2_–AgCl mixture (d–i), Si‐NW obtained from the electrolysis of solid SiO_2_ (j–n), and Si‐NW/Ag obtained from the electrolysis of solid SiO_2_–Ag mixture (o–s); (d), (j), and (o) are SEM images; (e), (g), and (k) are TEM images; (f), (p), and (q) are HAADF‐STEM images; (h), (i), (m), (n), (r), and (s) are element mappings of Ag and Si; (d–s): electrolysis voltage: 2.2 V; time: 8 h. Temperature: 850 °C; NaCl–CaCl_2_ molten salt.

Constant‐voltage electrolysis is conducted, with the cell voltage between the solid cathodes and graphite anode being at 2.2–2.6 V to avoid the formation of Ca‐based alloys.^[^ ^31^, ^32^, ^33^, ^34^, ^35^, ^36^, ^37^, ^38^, ^39^, ^40^, ^41^ ^]^ The cathode potential (versus Ag/AgCl) was monitored in one case during constant‐voltage electrolysis at a cell voltage of 2.2 V (Figure S1, Supporting Information), which shows that the cathode potential (versus Ag/AgCl) during the two‐electrode‐electrolysis process is consistent with that in the CV curves in Figure 2a, further verifying the preferential reduction of AgCl and formation of Ag–Si.

The current–time curves during the molten salt electrolysis also point to the formation of Ag–Si. No spike current is observed in the current–time curve for the electrolysis of SiO_2_ (grey curve in Figure 2b, labeled as Si‐NW), revealing that the reduction of sole SiO_2_ cannot result in a spike current. The spike current is observed in the current–time curve of the electrolysis of SiO_2_+Ag (blue curve in Figure 2b, labeled as Si‐NW/Ag). In this case, there is no reduction reaction of AgCl, therefore the spike current is also not attributed to the reduction of AgCl alone. The spike of the reduction current should be caused by the formation of Ag–Si. Compared with the electrolysis of SiO_2_–Ag, the spike appears in an earlier stage for the electrolysis of SiO_2_–AgCl (red curve in Figure 2b, labeled as Si‐NT/Ag), indicating much easier formation of Ag–Si alloy in the SiO_2_–AgCl case.

According to the Ag–Si phase diagram (Figure S2, Supporting Information), the Ag–Si alloy turns from solid to liquid (illustrated as stage 1 in Figure 2c) with the content of Si reaching 10 mol% at the electrolysis temperature of 850 °C. The liquid Ag–Si could induce the formation of Si‐NT by the liquid–solid mechanism via an oversaturation‐promoted precipitation of Si along the high‐energy edges of liquid Ag–Si clusters (illustrated as stage 2 in Figure 2c).^[^ ^20^, ^21^ ^]^ Generation of Si‐NT is verified by the scanning electron microscopy (SEM, Figure 2d) and transmission electron microscopy (TEM, Figure 2e) images. The corresponding powder X‐ray diffraction (XRD) pattern is indexed to Si and Ag (Figure S3, Supporting Information, denoted as Si‐NT/Ag). The corresponding high‐angle annular dark‐field scanning transmission electron microscopy image (HAADF‐STEM, Figure 2f), high‐resolution TEM image (Figure 2g) and energy‐dispersive X‐ray spectroscopy (EDS) elemental mapping images (Figure 2h,i) also confirm the generation of Si‐NT upon electrolysis of SiO_2_–AgCl.

Compared with the electrolysis of SiO_2_–AgCl, the sample obtained from the electrolysis of SiO_2_ (Figure 2j–n) consists of solid Si nanowires (Si‐NWs) instead of Si‐NT, verifying the critical role of Ag species on the formation of Si‐NT. Nanotube is also absent in the sample derived from the electrolysis of SiO_2_–Ag (Figure 2o–s), revealing that only the in situ deposited Ag can induce the formation of Si‐NT.

Formation of liquid‐phase intermediates is a critical factor to prepare Si‐NT via the liquid–solid mechanism.^[^ ^20^, ^21^ ^]^ In the case of SiO_2_–Ag electrolysis, the pre‐added Ag particles are mainly adhered to the surface of electrolysis‐generated Si‐NWs (Figure 2p–s). Therefore, the content of Si is high in the Ag–Si micro‐zones, which makes the composition of these Ag‐containing micro‐zones far away from the liquid Ag–Si regions (with an Ag content of ≈90 mol%, as illustrated in the red zone in Figure 2c and Figure S2, Supporting Information). For the electrolysis of SiO_2_–AgCl, AgCl is preferentially electro‐reduced to Ag (Figure 2a) and tightly adhered on the surface of Ni substrate, followed by subsequent deposition of Si on the surface of Ag. Therefore, Si is formed at Ag‐rich sites in this case, which facilitates the occurrence of liquid Ag–Si to direct the growth of Si‐NT via the liquid‐solid mechanism.

**Figure** **3**a–d show that the current collector substrates (Ni or Mo) are of metallic luster after electrolysis of SiO_2_–AgCl. Residence of Ag layers on the substrates is then verified (Figure 3e–h). The precipitated Si from the Ag–Si alloy gets separated from the substrates after HCl leaching, resulting in relatively light color in the mapping image of Si on substrate (Figure 3h). The time‐dependent electrolysis shows that Ag is first generated and formation of massive Si occurs after 4 h (Figure S4, Supporting Information). The content of Si increases with the electrolysis time, as evidenced by the increased diffraction intensity of Si in the XRD patterns of cathodic products (Figure S4, Supporting Information). After electrolysis, clear peaks relating to Ag are detected in the XRD pattern of Ni substrate, revealing the adherence of Ag on the Ni substrate (Figure S5, Supporting Information). Ag tends to stay on the Ni substrate, agreeing well with the absence of Ag along the Si‐NT (Figure 2i).

**Figure 3:**
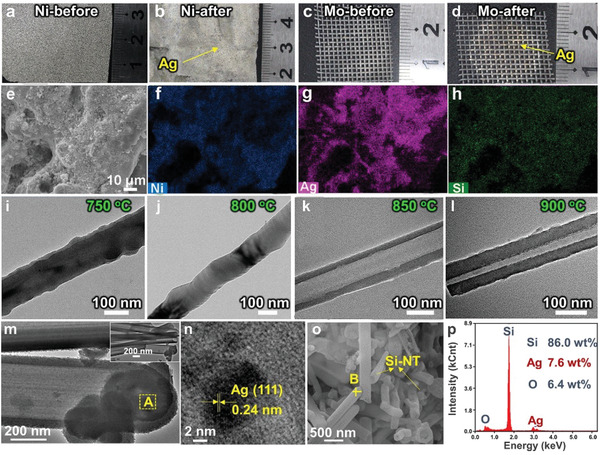
a–d) Optical images of Ni (a,b) or Mo (c,d) substrates before (a,c) and after (b,d) the electrolysis of SiO_2_–AgCl mixture in molten NaCl–CaCl_2_ at 850 °C; e) SEM images of the Ni substrate after electrolysis and f–h) the corresponding elements distribution images; i–l) TEM images of cathodic products obtained from electrolysis of SiO_2_–AgCl mixture at different temperatures at 2.2 V for 8 h. m,n) TEM and o) SEM images of Si‐NT/Ag; (n) is the magnification of area A in (m); p) the EDS spectra of site B in (o). The sample in (m–p) is obtained by electrolysis for 6 h at 2.2 V in 850 °C NaCl–CaCl_2_ molten salt.

A liquid phase is critical for the formation of silicon nanotubes or carbon nanotubes via the conventional vapor–liquid–solid (VLS) mechanism.^[^ ^19^, ^20^, ^21^, ^23^ ^]^ Another feature for the VLS mechanism is that liquid droplet is usually found in the tip of nanotube.^[^ ^19^, ^20^, ^21^, ^23^ ^]^ The assistance of liquid Ag–Si clusters on the formation of Si‐NT via the similar liquid–solid mechanism in Figure 1 is elaborately validated. First, the Ag–Si phase diagram demonstrates that the liquid Ag–Si can be generated during electrolysis (Figure 2c and Figure S2, Supporting Information), as evidenced by the fact that the electrolysis temperature (850–900 °C) is higher than the eutectic point (823 °C) of Ag–Si; second, TEM images show that silicon nanotubes can be only obtained at temperatures exceeding 823 °C (Figure 3i–l), further validating the suggested liquid–solid mechanism in Figure 1. Finally, spherical Ag–Si droplet in the tip of silicon nanotube is observed (Figure 3m), with the Ag (111) interplane distances (0.24 nm) being clearly detected (Figure 3n). The EDS results also validate the existence of Ag–Si in the tip area of silicon nanotube (Figure 3o,p). Compared with electrolysis temperature, the influence of electrolysis voltage (2.2–2.6 V) on the formation of Si‐NT is minor (Figure S6, Supporting Information). Electrochemical reduction of SiO_2_ is incomplete with short electrolysis time, resulting in only a small part of silicon nanotubes (Figure S7, Supporting Information). Electrolysis of the SiO_2_–AgCl mixture at 2.2 V for 8 h at 850 °C is the optimized condition for the preparation of Si nanotubes. For comparison, Si nanotubes can hardly be obtained from electrolysis of SiO_2_–Ag mixture at varied voltages (Figure S8, Supporting Information).

For the electrolysis of SiO_2_–AgCl, a vast majority of deposited Ag is closely adhered onto the substrates (Figure 3a–h and Figure S5, Supporting Information) due to a strong interaction between Ag and Ni (Figure S9, Supporting Information). The content of Ag in the obtained Si‐NT/Ag is negligible (<0.1 at%, see Figure S10, Supporting Information). Even a low content of Ag is added, the diffraction peaks of Ag in Figure S10, Supporting Information is strong, which is possibly caused by the much higher XRD cross‐section of metallic Ag than that of Si. The content of Ag determined from X‐ray photoelectron survey spectra (XPS, Figure S11a, Supporting Information) is only 0.02 at%, further validating the negligible content of Ag in Si‐NT/Ag. For comparison, the content of Ag in the Si‐NW/Ag obtained from the electrolysis of SiO_2_–Ag mixture is much higher, as evidenced by a much stronger intensity of peaks relating to Ag in both XRD patterns (Figure S3, Supporting Information) and XPS (Figure S11b,c, Supporting Information). During electrolysis of SiO_2_–Ag mixture, no liquid Ag–Si phase is formed and the pre‐added Ag mainly exists as solid particles (Figure 2p–s), making the capture of Ag by Ni substrate a tough task. Therefore, separation of Si from the pre‐added Ag particles is difficult. For the electrolysis of SiO_2_–AgCl mixture, the formation of flowable liquid Ag–Si phase can further promote the capture of Ag by Ni substrate. No intermetallic Ag–Si compounds exist (Figure 2c and Figure S2, Supporting Information), which means that the liquid Ag–Si alloy tends to be converted to separate phases of solid Ag and Si during the cooling process (stage 3 in Figure 2c), contributing to the self‐separation of Ag and Si.

Interestingly, when saturated AgCl is put into the NaCl–CaCl_2_ molten salt, the electrolysis of Ni‐wrapped SiO_2_ pellets results in Ag‐coated Si nanotubes (Si‐NT@Ag), presenting a facile in situ surface modification of Si nanotubes. As shown in **Figure** **4**a,b, the outer surfaces of Si‐NT are wrapped by a furry coating layer, which is verified to be Ag according to the element distributions of Si and Ag (Figure 4c–e). The existence of Ag layer is further validated by the high‐resolution TEM images taken from the surface area of Si‐NT@Ag (Figure 4f–i), as evidenced by distinct interlayer spacings of 0.24 nm, which is attributed to the Ag (111) crystal planes.

**Figure 4:**
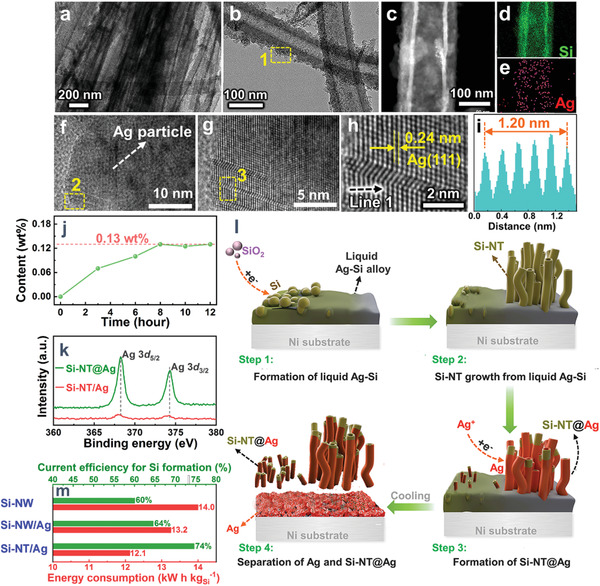
a,b,f–h) TEM, c) HAADF‐STEM, and d,e) the corresponding elements distribution of Si‐NT@Ag; (f), (g), and (h) are the magnifications of the dashed square area 1, 2, and 3, respectively; i) the interlayer distance profile along Line 1 in (h); j) dissolution curves of AgCl in NaCl–CaCl_2_ molten salt at 850 °C; k) XPS in Ag 3d regions. l) Illustration on formation of Si‐NT@Ag; m) current efficiency and energy consumption (electrolysis only) on preparation of different samples. Si‐NW and Si‐NT/Ag are obtained from the electrolysis of solid SiO_2_ and SiO_2_–AgCl mixture in 850 °C NaCl–CaCl_2_ molten salt, respectively. Si‐NT@Ag is obtained from electrolysis of solid SiO_2_ in AgCl‐saturated NaCl–CaCl_2_ molten salt at 850 °C. Electrolysis was conducted at 2.2 V for 8 h

The solubility of AgCl in 850 °C NaCl–CaCl_2_ molten salt is measured to be 0.13 wt% (Figure 4j), which means that the soluble AgCl near the outer surfaces of Si nanotubes can be electro‐reduced to Ag. The dissolution of AgCl is sluggish, which takes up to several hours to reach the dissolution equilibrium (Figure 4j). For the electrolysis of solid SiO_2_–AgCl pellets, the solid AgCl in cathode can be swiftly reduced to Ag before its dissolution into the molten salt, contributing to the alloying process for generation of liquid Ag–Si. In the case of dissolution‐saturated AgCl in the molten salt, the electrodeposition of Ag can take place preferentially in the initial stage in the electrochemical interface to trigger generation of Si‐NT. The potential difference between reduction of AgCl and SiO_2_ is only ≈0.2 V (Figure 2a). With the depletion of AgCl in the electrochemical interface, concentration polarization for electrochemical reduction of AgCl gets aggravated, which makes the deposition potential of Ag negative shifted, triggering the simultaneous electrodeposition of Ag on the surface of Si nanotubes and electrolysis of SiO_2_ in the late stage. The in situ coating of an Ag layer over Si nanotubes is hence achieved, as evidenced by the enhanced Ag 3d signals in the XPS (Figure 4k, 0.15 at% Ag for Si‐NT@Ag and 0.02 at% Ag for Si‐NT/Ag). Formation of Si nanotubes in AgCl‐saturated NaCl–CaCl_2_ molten salt undergoes a similar way with that in the electrolysis of SiO_2_–AgCl mixture (step 1 and step 2 in Figure 4l), but with extra Ag electrodeposited on the outer surface of Si nanotubes in the late stage (step 3 in Figure 4l). The separation between Ag and Si‐NT@Ag then occurs (step 4 in Figure 4l).

The electrode kinetics for molten salt electrolysis of solid silica for Si extraction is governed by ohmic polarization and mass transfer of/through the solid cathode.^[^ ^31^, ^32^, ^33^, ^34^, ^35^, ^36^, ^37^, ^38^, ^39^, ^40^, ^41^ ^]^ Electrolysis of SiO_2_–AgCl for Si formation ranks among the highest performance in terms of current efficiency (74%, Figure 4m) and energy consumption (12.1 kW h kg_Si_^−1^, Figure 4m). This energy‐consumption value is even lower than that of industrial production of metallurgical‐grade silicon (>20 kW h kg_Si_^−1^),^[^ ^41^ ^]^ promising future industrial production of Si nanotubes. Ag can increase the electric conductivity of the solid cathode. The Ag‐assisted generation of hollow structure can enhance mass transfer through the solid cathode. The mass of recycled Ag is determined to be 77–80% by comparing the weight difference of Ni substrate before and after electrolysis. The present protocol hence provides an intensified silicon extraction in terms of production yield and functionality of Si. Of note, molten salt electrolysis, which is widely adopted for production of metals in industry, has been verified to be adaptable for large‐scale production of silicon nanomaterials.^[^ ^31^, ^32^, ^33^, ^34^, ^35^, ^36^, ^37^, ^38^, ^39^, ^40^, ^41^ ^]^ Compared with the conventional CVD method which uses toxic SiH_4_/SiCl_4_ as raw materials and suffers from low production yield,^[^ ^12^, ^17^, ^18^, ^19^, ^20^, ^21^, ^22^, ^23^, ^24^, ^25^, ^26^, ^27^, ^28^ ^]^ the molten salt electrolysis strategy with a high production yield herein uses affordable SiO_2_ as the starting material and shows a promise for practical production of Si nanotubes.

The lithium storage capability of as‐prepared Si‐NT/Ag (obtained from the electrolysis of SiO_2_–AgCl in 850 °C NaCl–CaCl_2_ molten salt at 2.2 V for 8 h) is compared with the Si‐NW/Ag (obtained from electrolysis of SiO_2_–Ag in the similar condition). As shown in Figure S12a, Supporting Information, the sloped current from 1.5–0.3 V (versus Li^+^/Li) in the first CV cycle of the Si‐NT/Ag electrode is attributed to the formation of a solid electrolyte interphase film (SEI),^[^ ^11^ ^]^ which disappears in the following cycles. The cathodic peaks at 0.15 and 0.1 V originates from formation of Li–Si alloys. In the subsequent anodic scan, Li–Si alloys are delithiated to amorphous Si at 0.35 and 0.55 V. The current intensity of Si‐NT/Ag electrode become stronger upon cycling, which indicated that there is an activation process for the lithiation/delithiation of Si, as evidenced by the increase trend of capacity in the initial cycles (Figure S12b, Supporting Information).^[^ ^11^ ^]^ After 100 cycles at 1000 mA g^−1^, the specific capacity of Si‐NT/Ag keeps a high value of 1351 mAh g^−1^ (Figures S12b and S13b, Supporting Information). The value of Si‐NW/Ag is only 693 mAh g^−1^ (Figures S12b and S13a, Supporting Information). After cycling for 100 cycles, the tubular structure of Si nanotubes still keeps intact (Figure S14c,d), indicating that the free spaces inside the hollow Si nanotubes can maintain the structure integrity upon cycling, therefore contributing to a higher capacity retention when compared with the solid‐core Si‐NW/Ag.^[^ ^3^, ^4^, ^10^ ^]^ However, volume swelling of the samples are still observed after cycling test (Figure S14a,b, Supporting Information), which should be one important reason for capacity fading. In addition, the initial current efficiency (ICE) of Si‐NT/Ag (81%, Figure S12b, Supporting Information) is much higher than that of Si‐NW/Ag (62%, Figure S13a, Supporting Information), revealing that the hollow structure of Si‐NT/Ag with more exposed specific surfaces can promote the swift formation of stable SEI film.^[^ ^2^, ^4^, ^5^ ^]^

The capacity of Si‐NT@Ag is further improved with the aid of the buffer Ag layer, as manifested in Figures S12b and S13c, Supporting Information. After 100 cycle at 1000 mA g^−1^, the capacity of Si‐NT@Ag still maintains a high value of 1780 mAh g^−1^, much higher than that of the Si‐NT/Ag (1351 mAh g^−1^) counterpart. The coated Ag layer can act as a buffer layer to mitigate the volume variation during cycling. The increased Ag content on the surface of Si nanotubes can decrease the charge transfer resistance, as revealed by a much smaller semicircle of Si‐NT@Ag than that of Si‐NT/Ag in the high‐frequency regions of electrochemical impedance spectra (Figure S15, Supporting Information). Therefore, the Si‐NT@Ag shows improved performance than that of Si‐NT/Ag during the cycling tests. In comparison with recently reported Si nanostructures prepared by the same molten salt electrolysis method, the Si‐NT@Ag shows an obvious superiority in capacity at similar cycling number and charge/discharge rate.^[^ ^11^, ^41^ ^]^ Specially, the Si‐NT@Ag also shows comparable/superior performance with Si nanostructures prepared by some other methods.^[^ ^1^, ^2^, ^8^, ^10^, ^12^, ^19^, ^22^, ^26^ ^]^ Even so, substantial improvement for the battery performance is needed before evaluation for a full cell. The present study mainly focuses on increasing the fabrication efficiency of Si‐NT in molten salts. Mitigation of the capacity fading will be explored in the future by in situ coating carbon on the surface of Si‐NT as a buffer layer or preparation of C/Si‐NT composite to further improve the battery performance.^[^ ^12^, ^13^, ^14^, ^15^, ^16^ ^]^

In summary, we report a direct electrochemical method in molten salts for template‐free preparation and in situ surface modification of Si nanotubes. By simply varying the introduction of solid AgCl or soluble AgCl in electrochemical reduction of solid silica, Si nanotubes and Ag‐coated Si nanotubes could controllably be prepared. Importantly, the in situ generated liquid Ag–Si governs the formation of hollow nanotubes via the liquid‐solid mechanism and the automatic separation of Ag from Si. Such a novel modulation of Si electro‐crystallization from a solid‐solid process to the liquid–solid mechanism breaks the limitation of molten salt electrolysis in tuning the morphology of cathodic products, which means that the prepared Si nanostructures can be expanded from solid‐core Si nanowires or Si nanoparticles to the hollow counterparts. The in situ coated Ag layer not only facilitates the reduction of SiO_2_ by improving the electron‐transfer efficiency during electrolysis, but also enhances the lithium storage capability of the electrolytic Si by maintaining the structure integrity during lithiation/delithiation cycling. A ton‐scale pilot on extraction of silicon by electrolysis of silica in molten salts has been successful in China.^[^ ^41^ ^]^ The present strategy increases the current efficiency, decreases the energy consumption and enhances the lithium storage capability of the electrolytic silicon, which might advance the novel extraction of silicon toward large‐scale application.

## Conflict of Interest

The authors declare no conflict of interest.

## Supporting information

Supporting Information
