# Supplementary material for: Template‐Free Electrochemical Formation of Silicon Nanotubes from Silica
Source: Adv Sci (Weinh). 2020 Jul 10;7(17):2001492. doi: 10.1002/advs.202001492 (PMC7507395; doi:10.1002/advs.202001492)
Supplement: Supplementary file 1 — Supporting Information [file ADVS-7-2001492-s001.pdf]

Supporting Information

Experimental details

Materials: $CaCl_2$ (anhydrous, 99% purity) and NaCl (anhydrous, 99% purity) were purchased from Shanghai Titan Scientific Co. Ltd. Graphite rod (99.995% purity), nano-SiO_2 (15-30 nm in size, 99.5% purity), AgCl (99.5% purity), Ag powder (99.95% purity), commercial nanostructured silicon powder (~200 nm in size, 99.8% purity) were purchased from the Aladdin (Shanghai, China). Porous nickel (99.9% purity) and molybdenum net (99.95% purity) were provided by Dongguan Zehui New Material Technology co. LTD. All the chemicals were used as received without further purification.

Molten salt electrolysis: NaCl (172.5 g) and $CaCl_2$ (327.5 g) were well mixed and then contained in an alumina cruicible (inner diameter, 70 mm; height, 150 mm). The alumina cruicible was then transferred into an alumina tube reactor and protected by a high-purity argon flow at 150 mL min^{-1}, followed by heating at 400 °C for 24 h to remove the residual moisture. After that, the NaCl-$CaCl_2$ salt was then heated to 850 °C and kept for half an hour to enable complete melting. A graphite anode (15 mm in diameter, 99.99% in purity) and a nickel plate cathode were adopted for pre-electrolysis at 2.6 V for 12 hours to remove the residual water and impurities in the molten salt. The preparation of SiO_2-AgCl pellet cathode was as follows. Firstly, SiO_2 and AgCl with a molar ratio of 10:1 were dispersed in ethanol, followed by vigorous agitation for 4 h at room temperature under the protection of a tin foil paper to avoid the decomposition of AgCl. After drying at 60 °C for 12 h, the well-mixed SiO_2-AgCl powder was obtained, which was then die-pressed at 0.4 MPa to obtain the SiO_2-AgCl pellets (0.6 g in mass; ~20 mm in diameter; ~3 mm in height). Finally, the SiO_2-AgCl pellets were tightly wrapped by a porous nickel or molybdenum meshes, which were used as the cathodes. By replacing the SiO_2-AgCl mixture with SiO_2-Ag mixture or pure SiO_2, SiO_2-Ag or pure SiO_2 pellets were also prepared by the same way. Without specific mentions, electrolysis was conducted at 2.2 V for 8 h using the above nickel/molybdenum-wrapped pellets as cathode and graphite as anode at 850 °C in the NaCl-$CaCl_2$ molten salt. For electrolysis of nickel-wrapped SiO_2

pellets in AgCl-saturated NaCl-$CaCl_2$ molten salt, excessive AgCl (1.2 g) was added into the molten salt and then kept for more than 12 h to enable thorough dissolution before electrolysis. After electrolysis, the cathodes were soaked in the de-ionized (DI) water for more than 12 h to complete dissolve the entrained chloride salts, enabling the separation of porous Ni and silicon powders at the same time. The obtained powders were then leached in 0.5 M dilute hydrochloric acid for 24 h. The silicon powders were then collected after centrifugation in DI water and ethanol for several times, followed by vacuum drying at 60 °C.

Calculation on current efficiency and energy consumption: The current efficiency (Q_E) and energy consumption (E_c) was calculated by the following equation:[1]

$$Q_E = \frac{\frac{m_{Si}}{M_{Si}} \times F \times n}{\int I \mathrm{d}t} \quad (1)$$

$$E_c = \frac{U \times \int I \mathrm{d}t}{m_{Si}} \quad (2)$$

where m_{Si} (g) is the mass of obtained Si and M_{Si} (28 g mol^{-1}) is the molar mass of Si. F (96485 C mol^{-1}) is the Faraday constant. n is the electron transfer number, which is 4 for SiO_2 reduction. I (A) is the electrolysis current. t (s) is the electrolysis time and U (2.2 V) is the electrolysis voltage.

Solubility test: The solubility of AgCl in NaCl-$CaCl_2$ molten salt was determined as follows. 2 g AgCl was die-pressed at 0.4 MPa into pellets and tighly wrapped by porous nickel, which was then kept at the bottom of alumina crucible. Then, 500 g well-mixuted NaCl-$CaCl_2$ salt was added to the alumina crucible. After that, the alumina crucible containing the salts was transferred to the furnace and heated to 850 ^{o}C. After kept for different hours, the salt (~1 g) on the upper surface of the melt was scooped out by a home-made stainless stell spoon. The obtained salt was dissolved into dilute nitric acid and the content was then analyzed by an inductively coupled plasma optical emission spectrometer (ICP-OES, Agilent 7500a).

Cyclic voltammogram tests in molten salts: Cyclic voltammogram (CV) of AgCl was recorded using an AgCl loaded molybdenum (Mo) cavity working electrode in the eutectic NaCl-$CaCl_2$ molten salt at 850 °C by an electrochemical workstation (CHI1140C, Shanghai Chenhua, China),[2]

with the graphite rod (15 mm in diameter, 99.99% in purity) as the counter electrode and home-made Ag/AgCl as the reference electrode.[2] Similarly, CV curves of SiO_2 and AgCl-SiO_2 mixture (molar ratio, 1:1) were obtained by the same way. For the background CV curve of NaCl-$CaCl_2$ molten salt, the bare Mo cavity bar was used as the working electrode. All the CV curves were obtained at a scanning rate of 5 mV s^{-1}.

Li-ion battery performance: The slurry was obtained by thoroughly mixing the powdery electrolysis products (Si-NW/Ag, Si-NT/Ag, and Si-NT@Ag), sodium carboxymethyl cellulose (CMC), poly(acrylic acid) (PAA), and acetylene black in deionized water.[3] The weight ratio of electrolysis product, CMC, PAA, and acetylene black is 70:5:5:20. The working electrode with loading of around 2.0 mg cm^{-2} was then prepared by blade-coating the above slurry onto the copper foil and vacuum-dried at 120 °C for more than 12 h. The CR-2016 type coin cells were fabricated in an Ar-filled glovebox with moisture and oxygen below 1 ppm. Li foils and Celgard 2400 membrane were used as the counter electrode and separator. A mixture of ethylene carbonate and diethyl carbonate (mass ratio, 1:1) containing 1 M $LiPF_6$ was used as the electrolyte. The CV tests and electrochemical impedance spectra (EIS) were recorded on a multichannel electrochemical workstation (Solartron analytical 1470E). The scanning rate of CV were 0.2 mV s^{-1}. The frequency for the EIS was between 10^5 and 10^{-2} Hz with 10 mV amplitude and no applied potential bias. The galvanostatic charge/discharge profiles were performed on a Land battery tester in the voltage range between 0.01-1.5 V (*vs*. Li/Li^+).

Characterizations: Crystallographic configurations of the products were investigated by X-ray diffraction (XRD) on Rigaku Miniflex600 at a scan rate of 4 ° min^{-1} with Ni filtered Cu Kα radiation (λ = 1.5406 Å). Morphology of the prepared materials was detected by field-emission scanning electron microscope (FESEM, Zeiss SIGMA) and transmission electron microscopy (TEM, Titan G 2 60-300). The composition and elemental mapping images of the samples were analyzed by energy dispersive X-ray spectroscopy (EDS, GENESIS 7000 and OXFORD IET 200) attached to TEM apparatus. X-ray photoelectron spectra (XPS) were collected on X-ray photoelectron spectrometer (ESCALAB250Xi, Thermo Fisher Scientific) with the results calibrated by C 1s (284.8 eV).

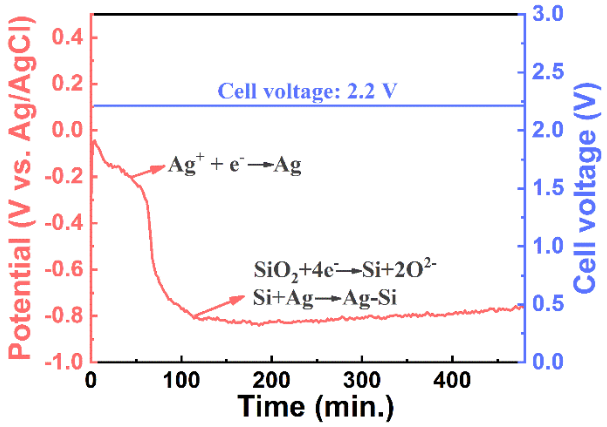

Figure S1. Cathode potential (vs. Ag/AgCl) monitored during constant-voltage electrolysis of SiO_2-AgCl mixture at a cell voltage of 2.2 V. Electrolysis temperature: 850 °C; Molten salt: equimolar NaCl-$CaCl_2$.

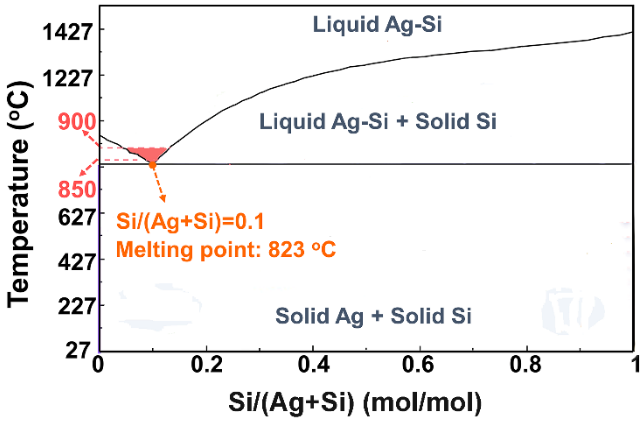

Figure S2. Ag-Si phase diagram. The electrolysis temperature for formation of Si-NT is 850-900 °C

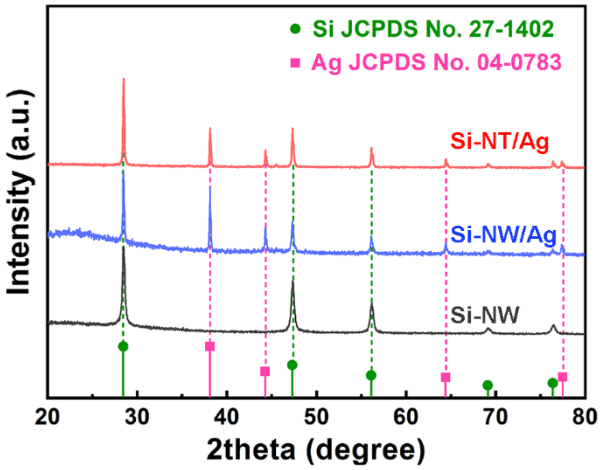

Figure S3. XRD patterns of the cathodic samples obtained from the electrolysis of SiO_2 (labeled as Si-NW), SiO_2-Ag (labeled as Si-NW/Ag), and SiO_2-AgCl (labeled as Si-NT/Ag) in 850 °C molten NaCl-$CaCl_2$ at 2.2 V for 8 h.

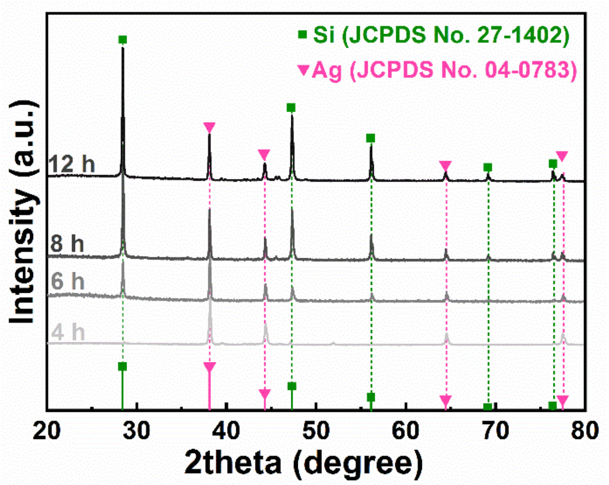

Figure S4. XRD patterns of the cathodic samples obtained from the electrolysis of SiO_2-AgCl in 850 °C molten NaCl-$CaCl_2$ at 2.2 V for different durations.

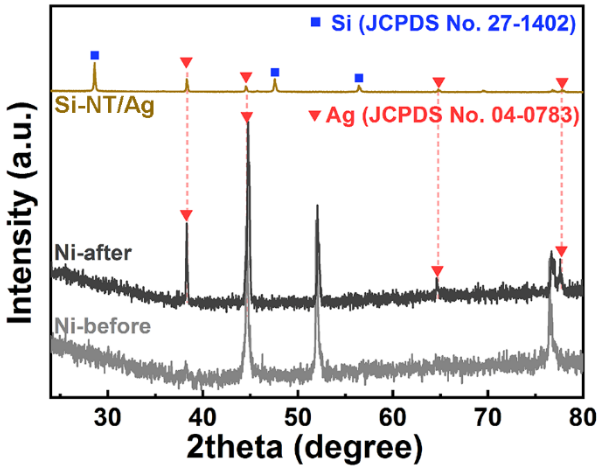

Figure S5. XRD patterns of Ni substrate before and after electrolysis of SiO_2-AgCl in 850 °C molten NaCl-$CaCl_2$ at 2.2 V for 8 h as well as the obtained Si-NT/Ag.

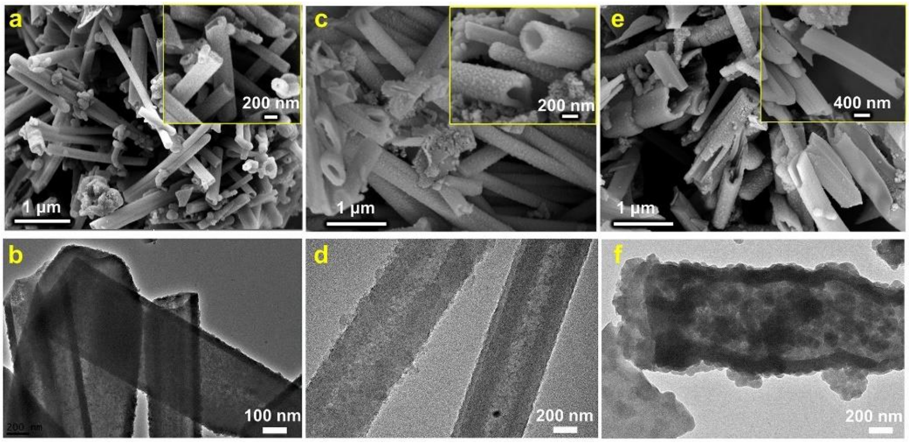

Figure S6. SEM (a, c, and e) and TEM (b, d, and f) images of the cathodic products obtained from electrolysis of SiO_2-AgCl in 850 °C molten NaCl-$CaCl_2$ for 8 h at 2.2 V (a, b), 2.4 V (c, d), and 2.6 V (e, f).

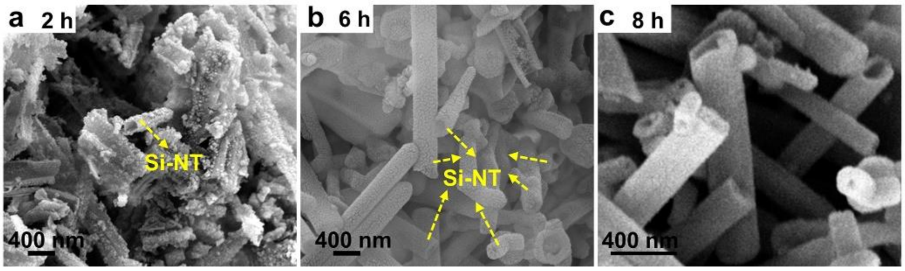

Figure S7. SEM images of electrolysis products obtained by the electrolysis of SiO_2-AgCl mixture at 2.2 V and 850 °C in NaCl-$CaCl_2$ molten salt: (a) 2 h; (b) 6 h; (c) 8 h.

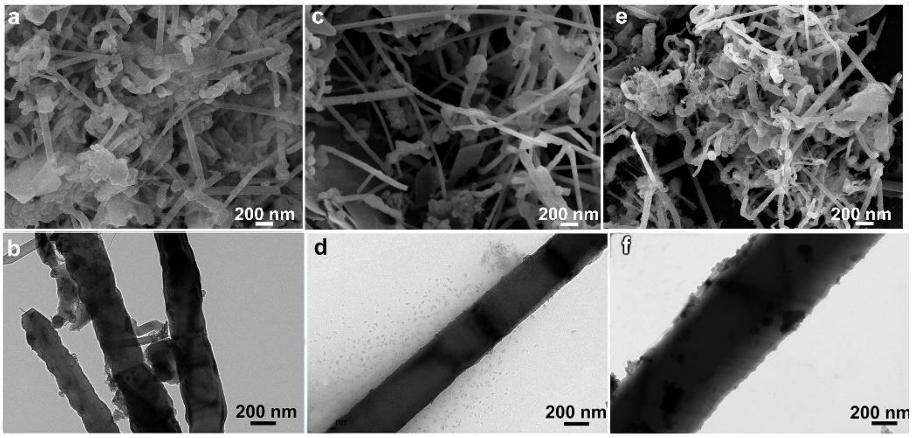

Figure S8. SEM (a, c, and e) and TEM (b, d, and f) images of the cathodic products obtained from electrolysis of SiO_2-Ag mixture in 850 °C molten NaCl-$CaCl_2$ for 8 h at 2.2 V (a, b), 2.4 V (c, d), and 2.6 V (e, f).

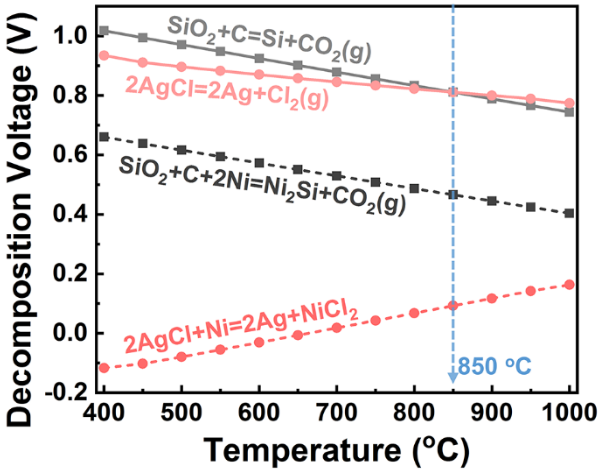

Figure S9. Decomposition voltages of some related reactions. Reaction between AgCl and Ni substrate to form Ag shows the lowest decomposition voltage, meaning a strong interaction between Ag and Ni substrate.

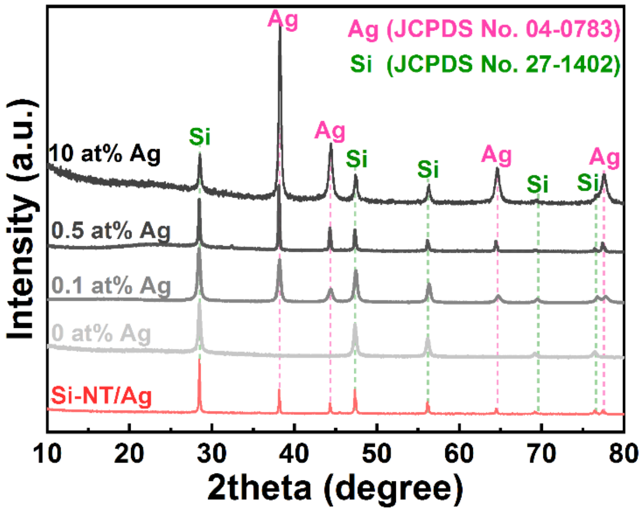

Figure S10. XRD patterns of various samples. Red line: Si-NT/Ag obtained from electrolysis of SiO2-AgCl mixture at 2.2 V for 8 h in 850 °C NaCl-$CaCl_2$ molten salt; Grey lines: commercial silicon powders containing physically mixed Ag (0-10 at% Ag). By comparing the peak intensity of Ag in Si-NT and factitious Ag-Si mixtures, the content of Ag in Si-NT is evaluated to be lower than 0.1 at%.

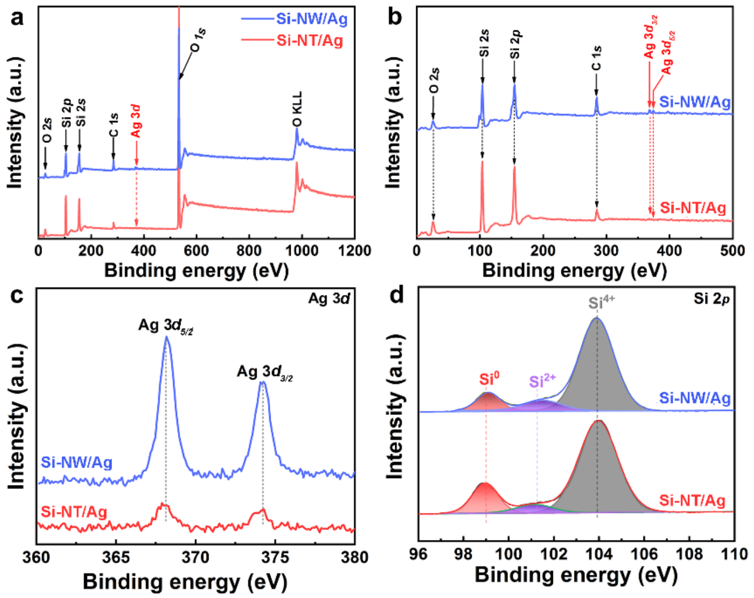

Figure S11. XPS results for the cathodic products obtained from electrolysis of SiO_2-Ag mixture (denoted as Si-NW/Ag) and SiO_2-AgCl mixture (denoted as Si-NT/Ag) in 850 °C molten NaCl-$CaCl_2$ at 2.2 V for 8 h. (a) Survey spectrum; (b) magnification of the survey spectrum; (c) Ag 3*d* spectrum; (d) Si 2*p* spectrum.

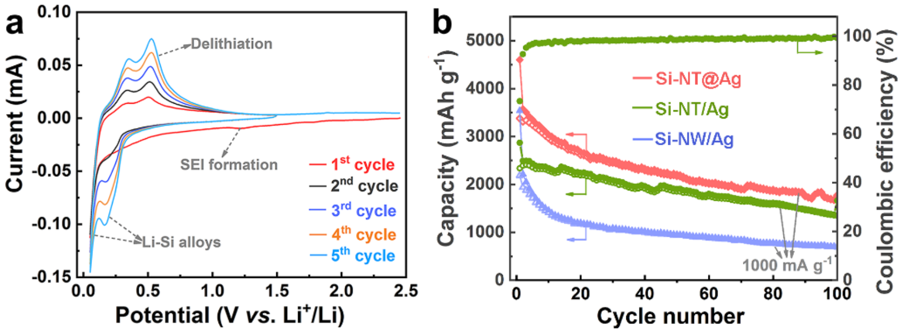

Figure S12. (a) CV curves of Si-NT/Ag electrode measured at 0.2 mV s^{-1}; (b) Cycling performance of Si-NW/Ag, Si-NT/Ag, and Si-NT@Ag as well as the Coulombic efficiency of Si-NT/Ag upon galvanostatic charge-discharge cycling at 1000 mA g^{-1}.

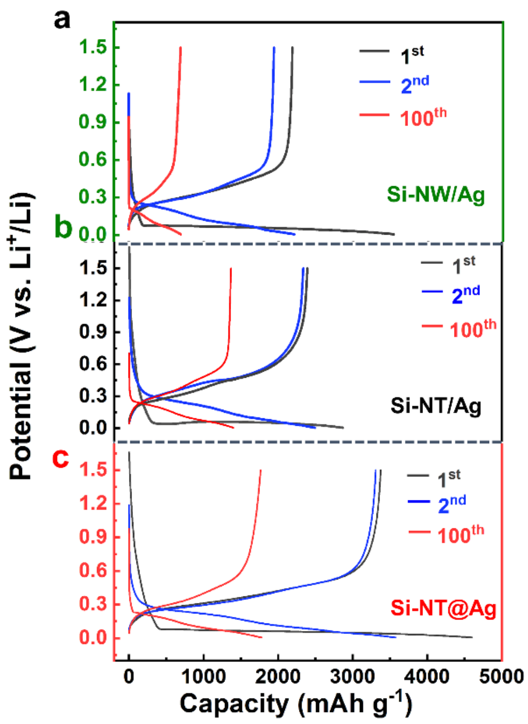

Figure S13. Discharge-charge curves of the Si-NW/Ag (a), Si-NT/Ag (b), and Si-NT@Ag (c) electrodes at 1000 mA g^{-1}. Si-NW/Ag and Si-NT/Ag are obtained by electrolysis at 2.2 V for 8 h in 850 °C NaCl-$CaCl_2$ molten salt from SiO_2-Ag and SiO_2-AgCl, respectively. Si-NT@Ag is obtained by electrolysis of SiO_2 at 2.2 V for 8 h in AgCl-saturated NaCl-$CaCl_2$ molten salt at 850 °C.

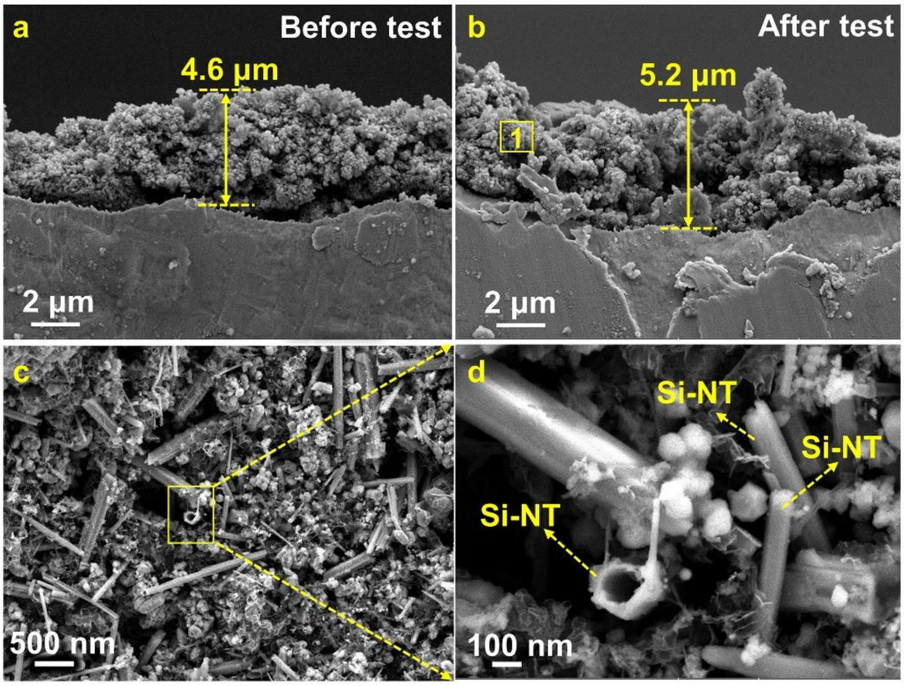

Figure S14. SEM images of the Si-NT/Ag electrode before (a) and after 100 cycles (b) at 1000 mA g^{-1}; (c) SEM images of the Si-NT/Ag electrode after 100 cycles and (d) the magnification of the squared area in (c). The loading of active material of the electrode is 0.8 mg cm^{-2}.

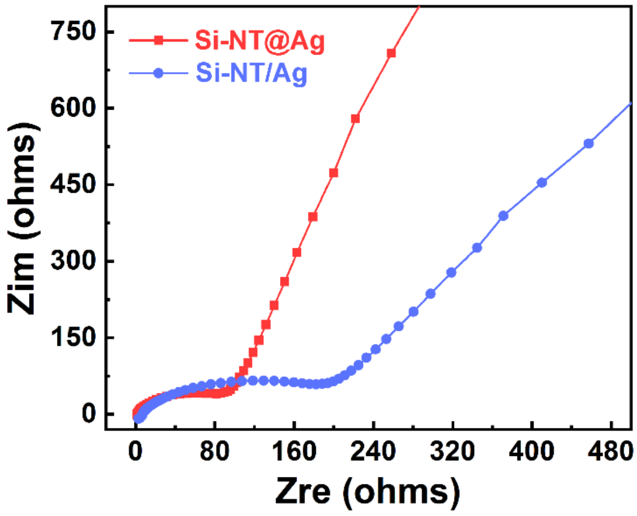

Figure S15. Electrochemical impedance spectra (EIS) of Si-NT/Ag electrode and Si-NT@Ag electrode obtained between 10^5 and 10^{-2} Hz.
